# The endothelial deprotection hypothesis for lupus pathogenesis: the dual role of C1q as a mediator of clearance and regulator of endothelial permeability

**DOI:** 10.12688/f1000research.6075.2

**Published:** 2015-05-11

**Authors:** József Prechl, László Czirják

**Affiliations:** 1 Diagnosticum Zrt, Budapest, 1047, Hungary; 2 MTA-ELTE Immunology Research Group, Budapest, 1117, Hungary; 3 Department of Rheumatology and Immunology, Clinic Center, University of Pécs, Pécs, 7632, Hungary

**Keywords:** lupus, SLE, systemic lupus erythematosus, pathogenesis, complement, C1q, autoimmunity, autoantibody, natural, IgM, endothelium, clearance, apoptosis

## Abstract

Systemic lupus erythematosus (SLE) is a heterogeneous multifactorial systemic autoimmune disease affecting several organs. SLE can start relatively early in life and results in impaired quality of life and shortened life expectancy because of a gradual disease progression leading to cardiovascular, renal and neoplastic disease. The basic mechanisms of the pathogenesis of the disease still remain to be clarified. It is clear that complement proteins play a key and complex role in the development of SLE. Complement component C1q has been known to be a fundamental component of lupus development, but most explanations focus on its role in apoptotic debris removal. Importantly, C1q was recently found to play a key role in the maintenance of vascular endothelial integrity.

We suggest that apoptotic products, endothelial cells and extracellular matrix components, which display negatively charged moieties, compete for binding to molecules of the innate humoral immune response, like C1q. Genetic or acquired factors leading to an increased load of apoptotic cell debris and decrease or absence of C1q therefore interfere with the regulation of endothelial permeability and integrity. Furthermore, we suggest that lupus is the net result of an imbalance between the two functions of immune clearance and vascular endothelial integrity maintenance, an imbalance triggered and sustained by autoimmunity, which skews C1q consumption by IgG-mediated complement classical pathway activation on autoantigens. In this triangle of innate clearance, autoimmunity and endothelial integrity, C1q plays a central role.

Hence, we interpret the pathogenesis of lupus by identifying three key components, namely innate immune clearance, autoimmunity and endothelial integrity and we establish a link between these components based on the protective role that innate clearance molecules play in endothelial renewal. By including the vasoprotective role of C1q in the interpretation of SLE development we attempt to provide novel explanations for the symptoms, organ damage, diagnostic and therapeutic difficulties of the disease.

## Introduction

**Figure 1.**
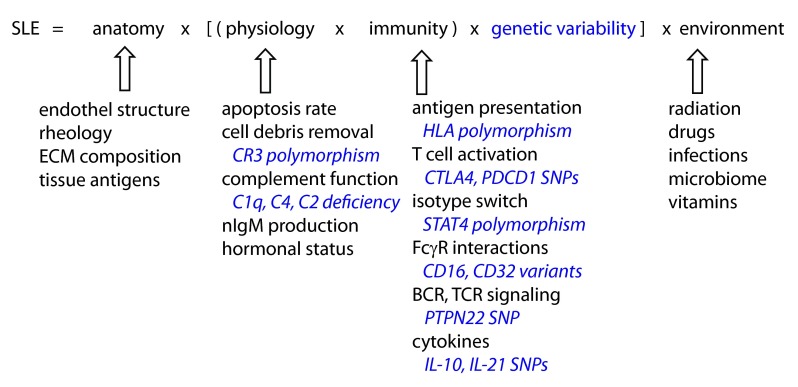
Systemic lupus erythematosus is a heterogeneous group of multifactorial diseases. The development of lupus is influenced by genetic factors, controlling individual variability especially with regard to the immune system and also with regard to physiology (some examples of genetic variability are shown in blue). Anatomical features may determine organ damage and source of autoantigens. Environmental factors can act upon all these elements, and may be the component we could try to modulate with the aim of preventing disease development. SLE, systemic lupus erythematosus; BCR, B-cell antigen receptor; TCR, T-cell antigen receptor; ECM, extracellular matrix; FcγR, Fc receptor for IgG.

A database search for the word “lupus” in the title of biomedical publications brings up 39,306 papers as of the writing of this manuscript. Thus, there is an abundance of experimental and clinical research data on systemic lupus erythematosus (SLE), yet the comprehensive etiopathogenesis of this group of heterogeneous diseases with multifactorial origin is still unknown ( Figure 1). SLE is an enigmatic disease, with a range of manifestations. Indeed, currently systemic lupus erythematosus is classified, but not diagnosed, on the basis of the coexistence of several alterations from a list of criteria ^1, 2^. Immune complexes containing IgG and complement are found deposited in various tissues and are responsible for inflammatory processes causing skin rash, mucosal ulcers, arthritis, nephritis, and serositis. Hematological changes include diverse cytopenias, while immunological tests show antibodies against nuclear material, dsDNA, Sm antigen and phospholipids ^3^.

In an attempt to simultaneously monitor antibody and complement binding to various autoantigens, we have developed a functional immunomics approach ^4, 5^ allowing a complex analysis of serological events in SLE. Interestingly, we observed that copious amounts of complement products are fixed by nucleic acids but not other negatively charged molecules in SLE patients with decreased complement C4 levels ^6^ (and manuscript in preparation), which finally led us to formulate the hypothesis presented below. In this paper, we attempt to collect all the pieces of knowledge of the lupus puzzle and place them next to each other in a way that a novel picture emerges. We hope that this hypothesis will stimulate discussions along a novel course and finally will result in a better understanding of the lupus syndrome.

## The pieces of the puzzle we already have

We can arrange most of the currently accepted mechanisms of lupus pathology in three main sets of factors: impaired clearance, autoimmunity and vascular injury. The elements of these particular processes are highly interconnected but for didactic reasons we will discuss them within one of these categories.

### Impaired immune clearance

***Increased load of cellular debris.*** The initiation of pathological autoimmune responses requires autoantigens to reach and trigger lymphocytes in the lymphoid organs. Cellular debris released from dying and dead cells is thought to be the most important source of self-antigen in lupus. One of the most widely used animal models of human SLE is the MRL/lpr mouse strain. These animals carry multiple susceptibility genes, which control lymphoproliferation and apoptosis ^7^, and spontaneously develop a lupus-like disease with antinuclear antibody production and nephritis. Interestingly, in human autoimmune lymphoproliferative syndrome (ALPS), where apoptotic signaling is impaired, some of the symptoms observed in SLE also appear ^8^, underlining the role of this factor. Direct evidence for the role of apoptotic load is also available, since increased apoptosis of monocytes ^9^, neutrophils ^10^, lymphocytes ^11^ and endothelial cells (EC) ^12^ has been described in SLE patients.

***Impairment of apoptotic debris removal.*** Cells undergoing programmed cell death are cleared from the body without inducing inflammation. This is part of the physiological tissue maintenance and regeneration events continuously occurring in the body. Dead cells, apoptotic blebs and debris are recognized by several soluble molecules and cell surface receptors, all promoting uptake by tissue macrophages and dendritic cells. This silent removal locally prevents inflammation and systemically the development of autoreactive lymphocytes ^13^. Inefficient removal of apoptotic cell debris in lupus ^14^ leads to the clonal expansion of autoreactive lymphocytes, with both B cells and T cells involved. Nucleosomes become accessible on the cell surface ^15^, exposing negatively charged nucleic acid containing complexes. NETosis, a form of programmed cell death recently described in neutrophil granulocytes, has also been implicated as a source of cellular debris that contributes to lupus pathogenesis ^16^.

The complement system plays an important role in apoptotic cell removal: C1q binds to negatively charged molecules like phosphatidyl serine and cardiolipin ^17^ and polyanionic targets, like DNA ^18, 19^. Various cells display receptors for C1q and help silent phagocytosis of apoptotic cells opsonized by C1q ^13, 20, 21^. The classical pathway of complement is activated, its components playing a hierarchical role in clearance ^22^. Deposited C4 and C3 fragments are then recognized by the CR3 of myeloid cells, a receptor encoded by lupus susceptibility gene ITGAM. The allelic variant of CR3 associated with lupus shows impaired phagocytotic and adhesion function ^23^.

***Hereditary complement deficiencies.*** Early complement components have long been known to play a key role in lupus development. Genetic deficiency of C1q is the strongest susceptibility factor for lupus ^24^, with close to 100% of the deficient subjects showing signs of the syndrome. Deficiency in the components involved in the later steps of classical pathway activation, C2 and C4, also predisposes to lupus development, albeit with lower probability. Interestingly, people with lupus show a secondary deficiency of these particular complement components suggesting the consumption of these proteins by factors playing a role in disease pathogenesis. These intriguing relations between complement and lupus have been discussed in depth by excellent reviews ^25, 26^.

***Complement deficiency due to consumption.*** Immunoglobulins undergo a conformational change upon antigen binding. This event coupled with immobilization and provision of affixed C1q binding sites promotes the binding of the C1 complex. The attachment of C1 will activate C1r and C1s, initiating the complement cascade ^27^. The fact that DNA specific immunoglobulins trigger complement activation to such an extent that the systemic consumption is measurable as decreased C4, C3 and CH50 levels has been known for decades ^28^. The measurement of these parameters forms part of the diagnostic routine even today, because secondary complement deficiency in lupus is associated with disease activity ^29^. Complement is also consumed by being deposited on blood cells in the circulation of lupus patients, a fact that is beginning to be exploited for diagnostic purposes ^30^.

### Development of autoimmunity

***Breaking of tolerance.*** Lymphocytes go through several checkpoints during their development, ensuring that self-reactivity is kept within a rational range ^31^. The process results in immunological tolerance to self-antigens. Tolerance can be broken by increased load of apoptotic cell debris reaching the secondary lymphoid organs ^32^, increased propensity for positive selection of B cells ^33^, the presence of molecular patterns in autoantigen that activates Toll-like receptors ^34^, the production of cytokines regulating B-cell development ^35^. The breaking of tolerance is characterized by the production of immunoglobulins specific for the autoantigens which induce autoimmunity, therefore these autoantibodies can be used as disease markers and also to identify the source of the autoantigens.

***Development of autoantibodies.*** Once tolerance against self is broken, antibodies against various nuclear components appear, including various forms of DNA, RNA, nucleosome complexes and nuclear proteins ^36^. Autoantibodies are detectable before the clinical onset of SLE ^37^ and with the development of organ damage various specific autoantibodies appear in the circulation ^38^. These can involve various extractable nuclear antigens ^39^, phospholipids ^40^, complement proteins ^41, 42^ and even cytokines like BAFF ^43^. The composition of the immune complexes has important consequences regarding its cell activating properties: DNA in the immune complexes that are formed upon the production of IgG antibodies stimulates plasmocytoid dendritic cells, which in turn release type I interferons, promoting tissue injury ^44^. The antigen-driven development and appearance of high affinity double-stranded DNA specific IgG is considered a hallmark of systemic lupus erythematosus ^45^.

### Vascular injury

***Circulating immune complexes bind to vessel wall.*** In lupus, immune complexes are found in the circulation, attached to vessel walls and deposited perivascularly. Binding to capillary wall endothelium was shown to be dependent on the presence of C1q in the immune complexes C1q ^46– 48^. Thus, once immunity is triggered against autoantigens, the presence of both autoreactive IgG and autoantigen in the circulation will lead to the formation of C1q containing immune complexes. Circulating immune complexes with characteristic components are found in various autoimmune diseases ^49^.

***Neutrophil granulocytes and FcγRs as effectors of inflammatory injury.*** Neutrophil granulocytes rolling along the vessel wall bind to the IgG component of deposited ICs via Fc gamma receptors. This binding, as modelled in our functional antibody assay ^50^, will trigger adhesion and activation of the cell. Neutrophil granulocytes secrete type I interferon and play important roles in the initiation and perpetuation of the disease ^51^. Fc gamma receptors displayed by the granulocyte play intricate roles in the recognition and uptake of IgG immune complexes and the induction of NETosis ^52^. Dysregulation of NET formation itself has also been suggested to play a role in lupus pathogenesis ^53^.

***Endothelial cell (EC) dysfunction in lupus.*** Lupus patients have a high risk of developing cardiovascular disease. Endothelial dysfunction, one of the key factors of atherogenesis, can be triggered by various endothelium damaging factors present in lupus ^54^. The effects of cytokines, inflammatory cells and immune complexes are combined with compromised endothelial functions resulting in increased atherogenicity in lupus ^54^. Endothelial repair is also compromised by a decrease in the number of bone marrow derived endothelial cell progenitors ^55^. Interestingly, C1q and mannose binding lectin (MBL), recognition molecules of the classical and lectin pathways of complement activation, respectively, help remove atherogenic lipoproteins ^56^, establishing a link between C1q deficiency and cardiovascular disease development in lupus. Increased vascular endothelial permeability resulting in edema, infiltration of inflammatory cells and deposition of immune complexes are the commonly observed histological features of the disease.

***Coagulation and thrombosis defects in lupus.*** SLE patients are susceptible to cardiovascular morbidity and mortality ^57^. Abnormal coagulation and thrombus formation is associated with the presence of anti-phospholipid antibodies, exemplified by anti-cardiolipin, anti-β2-glycoprotein and lupus anticoagulant antibodies ^58^. These autoantibodies have been suggested to directly cause endothelial injury or promote atherogenesis by altering lipoprotein metabolism ^59^.

## The missing piece: molecules of innate immunity contribute to endothelial integrity

One important question that has been left largely unanswered so far is why and how exactly are the vascular wall and surrounding tissues damaged? How do the above named three main categories, impaired clearance, autoimmunity and vascular injury interact with each other? We propose that the answer lies in the protective role that innate clearance molecules play in endothelial renewal.

**Figure 2.**
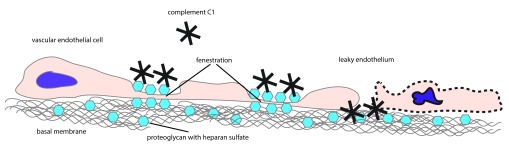
Interactions of C1q with the endothelium. Complement C1q binds to the negatively charged heparan sulfate-rich proteoglycans of the glycocalyx. At leaky endothelial junctions C1q will bind to the subendothelial basal membrane, where it promotes adhesion and regeneration.

The globular head of complement C1q binds to negatively charged molecules like DNA ^60^ and cardiolipin ^17^, and also to immunoglobulins mainly via ionic interactions ^61^. C1q has also been shown to bind heparan sulfate ^62^, a component of the extracellular matrix and of the surface structures of adherent cells ^63^. Thus, C1q beyond its role in the clearance of apoptotic cell debris could also be involved in endothelial cell interactions with the extracellular matrix. Indeed, C1q has been shown to play a role in vascular regeneration by binding to endothelial cells and promoting endothelial adhesion and spreading ^64^ and exert proangiogenic effects like stimulation of endothelial proliferation, migration and permeability ^65^. With these multiple roles of C1q in mind, we propose the following two scenarios, one describing events occurring in the presence of intact endothelium and another for leaky endothelium ( Figure 2).

### Proposed role of C1q in transendothelial transport

In a healthy adult male, the endothelium is quiescent ^66^. Significant amounts of C1q bind to EC only in tissues with discontinuous endothelium. Discontinuous endothelium is found at sites of transendothelial trafficking and is characterized by the fusion of the luminal and abluminal plasma membranes, the presence of pores of various diameters and high heparan sulfate content of the glycocalyx ^67, 68^. Discontinuous endothelium is divided into sinusoidal and fenestrated types. Sinusoidal endothelium lines bone marrow sinuses, splenic and liver sinuses; fenestrated endothelium covers capillary walls in kidney glomeruli, in the gastrointestinal tract, in endocrine glands and in the choroid plexus ^66^. In healthy adult females of the childbearing age the endothelium is subject to the effects of factors that regulate cyclic renewal of the endometrium, an event accompanied by vascular regeneration ^69^.

Where endothelium is discontinuous the glycocalyx becomes a major determinant of transendothelial traffic. The glycocalyx is a layer of macromolecules, mostly glycosaminoglycans, decorating the surface of ECs. Heparan sulfate constitutes more than 50% of the glycosaminoglycan pool in EC, localizing especially in cave-like structures (caveolae) ^70^ and the fenestrae ^67^, both areas playing primary roles in transendothelial transport and filtration, respectively. We envisage that C1q binds to heparan sulfate-rich regions on the luminal surface of EC and is also efficiently transported to the abluminal side into the tissues.

### Proposed role of C1q in paracellular endothelial leakage

If the integrity of the endothelium is disrupted, the subendothelial lamina becomes exposed to the blood plasma. When ECs are in the process of cell division or death, large pores with diameters reaching 1 micrometer are formed in the endothelium, as a result of cellular discontinuity ^71^. Upon exposure, subendothelial collagen immediately binds to several molecules from the blood plasma, triggering repair, coagulation and thrombocyte binding. This event remains silent as long as the endothelium is only modestly damaged. Even though the physiological turnover of EC is low ^72^ subendothelial collagen can be exposed whenever and wherever endothelial cells die. The renewal process is restricted in time and space, unless massive endothelial cell apoptosis is triggered by external factors, such as UV radiation. C1q or C1q containing IC can pass through these leaky junctions and deposit in the extracellular matrix (ECM), where heparan sulfate is an essential proteoglycan component ^73^. Since C1q is an eat-me-silently signal for myeloid cells, as discussed above, C1q deposition protects these areas of endothelial regeneration from myeloid cell-mediated damage, until integrity is reconstituted.

Based on the above observations we hypothesize that sufficient amounts of free C1q should be available in the blood in order to maintain endothelial trafficking, integrity and renewal. Free serum C1q protects exposed collagen from triggering attachment, activation and extravasation of monocytes and neutrophil granulocytes during endothelial renewal.

### Potential role of other molecules with similar binding pattern

However, C1q is actually not the only multivalent molecule which binds negatively charged moieties. In addition there is – at least – one other molecule with the ability to bind to apoptotic cells multivalently. Natural IgM (nIgM) molecules, which are produced without a clearly identifiable antigenic stimulus, have been shown to promote the clearance of apoptotic cells in mice ^74^ and enhance phagocytic clearance of host cells ^75^. IgM against dsDNA was shown to be protective in a murine autoimmune model ^76^. The general immunological protective properties of natural IgM were recently reviewed by Grönwall *et al.* ^77, 78^. Anti-apoptotic cell IgM antibodies bind C1q and promote clearance by phagocytes ^79^. An interesting aspect of IgM is that it can bypass the classical activation pathway by binding MBL and induce C4b deposition via MASPs. MBL deficient mice displayed impaired apoptotic cell clearance, without overt signs of autoimmunity, suggesting an alternative role for clearance by the lectin pathway ^80^. Natural IgM binding and the ensuing events lead to the recognition and removal of apoptotic cells without activating the phagocytic cells ^75^. The fact that antibodies of the IgM class do bind to collagen and this binding is decreased in lupus patients has been reported ^6, 81^. The question that remains to be answered is whether it is nIgM that binds to collagen. It is also intriguing whether other molecules of the innate humoral immune system, such as pentraxins and collectins may play similar roles.

In summary, multivalent molecules with a propensity to bind negatively charged targets and the ability to initiate complement activation are consumed by apoptotic cells, immune complexes, EC and the extracellular matrix. As long as these molecules are available in excess this competition will go unnoticed. Once this balance is tipped pathological events start to take place.

## Putting the pieces together: the endothelial deprotection hypothesis

**Figure 3.**
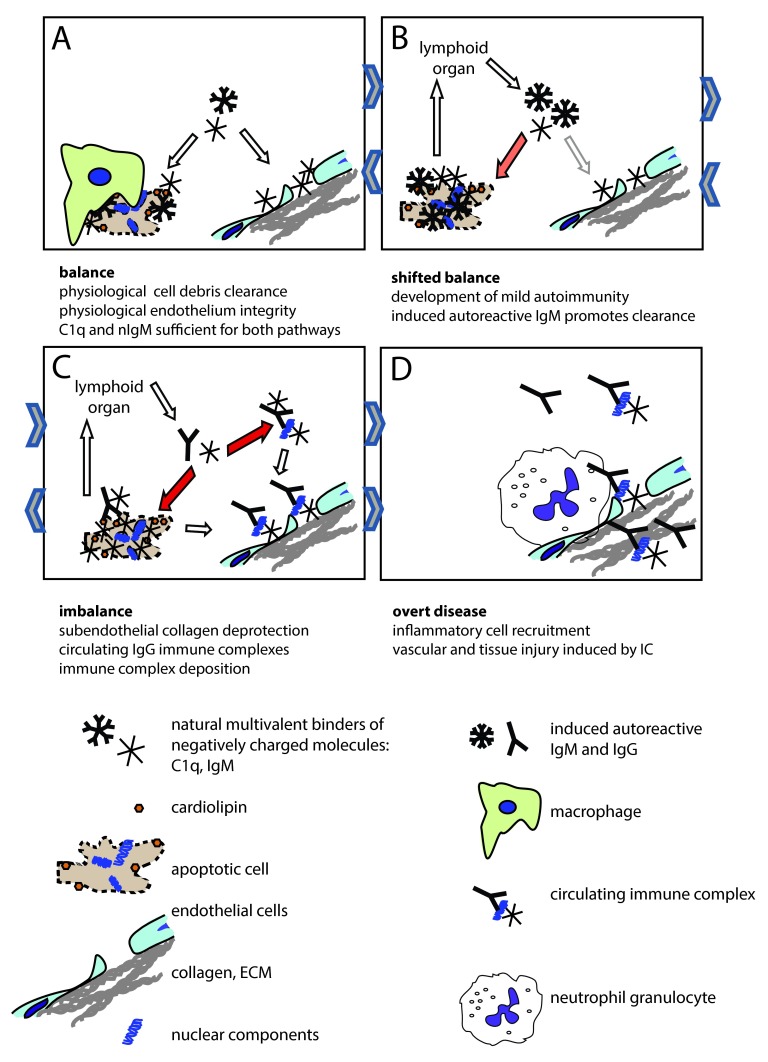
The endothelial deprotection hypothesis. Maintenance of endothelial integrity and clearance of cellular debris both requires multimeric innate molecules (C1q, nIgM) with the ability to bind anionic surfaces ( **A**). C1q binds to fenestrated regions of the endothelial cell and to collagen exposed due to leaky endothelial junctions. Increased use of these innate molecules by the clearance mechanism, or deficiency of these molecules shifts this balance, which results in cellular debris reaching the lymphoid organs ( **B**). This triggers the production of IgM against the autoantigens. Induced autoreactive IgM binds to apoptotic autoantigens, activates the complement system and promotes clearance. Sustained autoantigenic stimulus and genetically determined clearance deficiency coupled with tendency of mounting inflammatory immune responses will result in the production of IgG against the tissue derived autoantigens ( **C**). Immune complexes containing IgG and C1q will bind to the vascular wall, since free C1q levels are low and circulating immune complexes will outcompete them. Deposited immune complexes containing IgG recruit white blood cells with Fcγ receptors, which can trigger cell activation, release of inflammatory cytokines, frustrated phagocytosis, NETosis ( **D**). Immune complexes and autoantibodies can penetrate the tissues via the damaged endothelium, causing organ specific damage. Sustained inflammation leads to irreversible organ damage. Orange and red arrows indicate mild and strong redirection of C1q usage, respectively. Arrows in between figure boxes indicate reversibility ( **A**– **C**) or irreversibility ( **C**– **D**).

If C1q binds negatively charged molecules then those molecules compete for C1q binding. Competition for C1q binding by deoxyribose and heparan sulfate has been experimentally confirmed ^62^. Thus, negatively charged components of the exposed nuclear content of dying cells and of the EC fenestrae or the exposed subendothelial matrix can all bind C1 complex from the circulation. Physiologically there is sufficient amount of C1 available and a balance exists between the vessel wall and cellular debris. This binding results in tightly controlled classical complement pathway activation, leading to the production of C4b. Other factors controlling complement activation also bind to negatively charged molecules; these include complement factor H, C4bBP ^82^ amongst several other recognition molecules of the innate humoral immune system, like pentraxins ^83^ and surfactant proteins ^84, 85^. These molecules regulate further activation of the complement cascade, the production of anaphylatoxins and the activation of surrounding and recruited cells. Opsonized apoptotic debris will therefore be silently removed by macrophages ( Figure 3A). The fact that not only the absence of C1q but also functional C1q deficiency leads to lupus development ^86^ suggests that initiation of the classical pathway activation is required for the removal of cellular debris and prevention of development of immune complex disease.

This balanced binding to EC, subendothelial collagen and cellular debris can be tipped basically by three main factors: decrease in C1q levels, increased load of apoptotic cells and increased collagen exposure. Genetic deficiency in C1q is accompanied by highly increased (more than 90%) likelihood of developing SLE ^87^. Increased cell death can be triggered by ionizing radiation or by drugs with cytotoxic effects ^88^. Inefficient phagocytic capacity of CR3 polymorphic variant r77h ^89^, a lupus susceptibility factor, may also increase apoptotic load. Increased collagen exposure can be the result of endothelial apoptosis induced by sunlight but physiological turnover of the endothelium is also accompanied by macromolecular permeability and access to the subendothelial lamina ^90^.

As a result of one or more of these factors, cellular debris will reach secondary lymphoid organs, triggering immunity against self-molecules, including DNA. IgM is first produced, which may help restore the balance by enhancing apoptotic debris removal via complement activation ( Figure 3B). This is in agreement with the findings of Li *et al.*, who reported increased IgM reactivity to several autoantigens in patients with incomplete lupus erythematosus syndromes ^36^. The generation of DNA specific IgG further tips the balance towards opsonization of cellular debris, taking away more C1q, leaving exposed subendothelial collagen unprotected ( Figure 3C). Furthermore, circulating complexes of nuclear material, IgG and C1q will bind to exposed collagen by nature of the multivalent C1q molecule and diffuse into the tissues. Alternatively, in organs with discontinuous endothelium, immune complexes will bind to the fenestrae and be transported into the tissue. Deposition of immune complexes containing IgG will trigger activation of monocytes and neutrophil granulocytes, attracted by C3 and C5 derived anaphylatoxins of the alternative pathway. This results in damage to the vessel wall itself, to increased permeability and to IgG-mediated damage to the tissues ( Figure 3D).

To summarize, impaired clearance of cell debris and immune complexes together with pathological anti-nuclear antibodies consume C1q, an important vascular regeneration factor, from the circulation, by directing it to immune complexes and apoptotic cells. In turn, not free but immune complex-bound C1q will attach to EC and exposed subendothelial collagen. IgG will trigger inflammation instead of regeneration. According to this scenario, dsDNA IgG triggers inflammation, while dsDNA IgM can act against it, by competing with IgG for dsDNA binding. Indeed, the ratio of dsDNA IgG to IgM has been shown to be a good indicator of renal damage in SLE ^91, 92^.

## Interpreting the lupus syndrome in light of the hypothesis

### Sex difference in susceptibility

Even though lupus can develop in both men and women, 90% of patients diagnosed with the disease are women, most of them being in the childbearing age ^93^. How hormones contribute to this skewed susceptibility is not defined. Our hypothesis emphasizes the role of vascular endothelial renewal in the pathogenesis of SLE, pointing to sex differences in angiogenesis.

In fact, angiogenesis is a critical component of endometrial renewal. Various hormones and growth factors interact during the formation of new vessels, including vascular endothelial growth factor (VEGF) ^69, 94^. VEGF is produced in ovarian tissues during the menstrual cycle and regulates vascular remodeling and repair. VEGF is a permeability factor as well, its topical administration can induce the development of fenestrations in the endothelium of small venules and capillaries ^95^. Estradiol itself can also directly increase permeability ^96^ and act indirectly ^97^ by modulating VEGF production in endothelial cells. As highlighted above and further discussed below, fenestration, accessibility of the subendothelial lamina promotes the deposition of both bare C1q and nIgM and C1q containing immune complexes. This would render women of the childbearing age, with functioning ovaries, more susceptible to immune complex deposition and vascular damage in SLE.

### Organ damage in SLE

**Figure 4.**
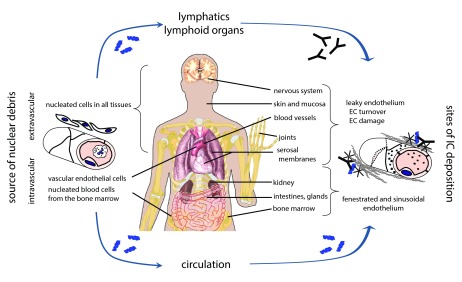
Endothelial deprotection and organ involvement in SLE. Apoptotic nuclear material can originate within the circulation or outside of the circulation. Apoptotic or apoptosis-prone nucleated blood cells can enter the blood stream from the bone marrow crossing the sinusoid endothelium. Endothelial cell apoptosis can be triggered by physical or pharmacological stimuli. These events result in circulating apoptotic debris load unless it is swiftly removed by physiological clearance pathways. Apoptotic debris outside of the circulation reaches the draining lymph nodes. Anti-nuclear antibody production is induced in the lymphoid organs when genetic and environmental susceptibility factors interact. Nuclear debris within the circulation is covered by various clearance molecules, including C1q. Anti-nuclear IgM and IgG modulates C1q deposition and complement activation. C1q promotes the deposition of nuclear immune complexes at sites where the endothelium is leaky or fenestrated. These surfaces are normally masked and covered by C1q and early complement activation products, along with other innate protection molecules, like nIgM. Overload of apoptotic debris can however consume these molecules, deprotecting the endothelium and opening the way for IgG Fc receptor mediated inflammation and tissue damage. While the endothelial lining of the liver is sinusoid, the lower blood pressure due to portal venous blood supply and the unique macrophages (Kupffer cells) of the liver seemingly prevent accumulation of IC. Source of body organ depictions: Wikimedia Commons.

Our hypothesis suggests that anatomical sites with discontinuous endothelium and tissues where collagen in the subendothelial lamina is exposed will be more vulnerable to immune complex induced damage. There is one more important aspect we have to consider: what is the source of the autoantigen that will induce autoimmunity? Keeping with the notion that in lupus it is the material from dead cells that induces autoimmune response we need to locate the source of apoptotic cells. Here we consider two main categories: cell death within the circulation and outside of the circulation ( Figure 4). Within the circulation it is the corpuscles in the blood ^98^ and endothelial cells ^12^ that are sources of cellular debris. Actually we will also consider the bone marrow as a source of apoptotic debris within the circulation because of the high rate of apoptotic death during lymphocyte development and the sinusoidal structure of the endothelium in the tissue. Apoptotic antigens within the circulation will be distributed by the blood flow throughout the body. Immune complexes formed from this material will be deposited anywhere where there is blood flow, and preferentially where blood flow is high, the endothelium is discontinuous and where subendothelial collagen is more accessible. We suggest that renal, bone marrow, joint, serosal and synovial, and partly skin damage in lupus is mediated by this route and constitutes the core components of SLE.

Apoptotic cells that come into contact with blood also initiate coagulation events ^99^. Therefore in addition to lipids that become exposed on apoptotic cells, phospholipid-binding proteins (β2-glycoprotein) and components of the coagulation cascade will serve as autoantigens targeted by the immune response. Anti-cardiolipin antibodies and lupus anticoagulant could be produced as the result of these pathological events ^100^, and are responsible for the secondary anti-phospholipid antibody syndrome in SLE.

Outside of the circulation, basically meaning in the tissues, apoptotic cells and their fragments and antigen presenting cells carrying and processing those will reach the secondary lymphoid organs first. Antibodies generated in the lymphoid organs will enter the circulation and immune complexes may or may not be formed, depending on the presence or absence of antigen. Once these autoantibodies appear, they could sustain organ specific damage and disease course by binding to their targets thanks to increased permeability by general impairment in endothelial regeneration.

***Photosensitivity.*** Cutaneous manifestations of SLE are often linked to exposure to sunlight or artificial sources of ultraviolet (UV) light. Malar rash, the butterfly shaped erythematous lesion on the face is a classical sign of lupus. It may be present in about 50% of SLE patients at the time of the diagnosis ^101^ and is part of the general photosensitivity observed in SLE. UV-light induced apoptosis of keratinocytes is thought to be a source of cellular debris that promotes the induction of rheumatic diseases ^102^. Actually endothelial cells are quite sensitive to radiation-induced, ceramide-mediated apoptotic cell death ^103– 105^. We speculate that UV light penetrating the epidermis may cause endothelial damage in dermal capillaries. Dead endothelial cells will be removed inefficiently in the relative absence of C1q and nIgM, while the exposed subendothelial lamina will be less protected by these molecules. Increased paracellular leakage and transcellular trafficking in activated EC would result in increased deposition of IC, edema and extravasation of myeloid cells and the appearance of rash. Additionally, the entry of apoptotic endothelial cells ^12^ or their products containing nuclear material and proinflammatory mediators ^106^ into the circulation may contribute to disease flares triggered by UV exposure.

***Renal involvement.*** SLE can lead to the development of lupus nephritis, which is one of the most disabling complications ^107^. The kidneys are prone to immune complex mediated damage for at least two reasons. It is the organ with the second highest blood flow rate ^108^ and the endothelium is fenestrated, leaving access to C1q bearing immune complexes. Apoptotic debris that is generated within the circulation or enters the circulation will have a very high chance of ending up in the glomeruli. Should these complexes contain IgG, the necessary component for triggering inflammation will be present. Other factors, such as DNAse activity may modulate the severity and prognosis of nephritis ^109^.

***Synovitis and serositis.*** Besides ionizing radiation mechanical injuries may also negatively influence endothelial integrity. Synovial and serosal membranes are rich with blood circulation and are continuously exposed to micromechanical injuries due to the movement of the joints and inner organs, respectively. We suggest that the healing of these microinjured sites would be slower and accompanied by edema and cellular infiltration, because of the deposition of circulating immune complexes. These microinjuries may therefore be responsible for arthritis, pleuritis and pericarditis in SLE ( Figure 4).

***Bone marrow involvement.*** The bone marrow is a site of intensive cell proliferation and cell death. Lymphocyte development involves selection steps when useless or harmful clones are deleted by programmed cell death. Any defect in the clearance of apoptotic cells is therefore expected to influence homeostasis in this tissue. Additionally, it is a site where there is intensive migration via the endothelial layer in both directions, facilitated by a special endothelial structure: the sinusoidal endothelium. We suggest that this tissue would be vulnerable to immune complex deposition and inflammation. In SLE patients abnormal bone marrow histology is observed ^110^. Comparative analysis of gene expression revealed upregulation of genes involved in cell death and granulopoesis in active SLE patients, confirming the role of apoptosis and granulocytes in the pathogenesis of the disease ^111^. We suggest that cytopenias, the detection of which constitute pillars of the diagnostic algorithm of SLE ^2^, are the consequence of abnormal bone marrow function, in addition to specific antibody mediated direct damage.

### Consequences for diagnostic efforts

Classification of systemic lupus erythematosus relies on clinical examination, hematological tests and serological test. The presence of dsDNA specific IgG is quite specific but less sensitive for the identification of SLE patients ^6^. Decreased complement levels and complement activity are also used but not specific for the disease. Indeed our hypothesis suggests that there is no single protein marker, which could be used alone, because a state of imbalance can only be assessed by the measurement of the different components characterizing the degree of imbalance. The measurement of free C1q or C1 complex and also of nIgM could be assessed for incorporation into the set of laboratory tests characterizing autoimmune conditions. Alternatively, instead of measuring the levels of individual proteins, a functional test that is capable of gauging the degree of endothelial damage, the propensity of immune complex deposition and inflammatory cell reactions all together, could be used for assisting diagnosis.

### Implications for future therapy and prevention of SLE

The causal therapy for lupus, based on the endothelial deprotection hypothesis, would be the restoration of endothelial integrity by the introduction or induction of C1q or nIgM. In the case of genetic C1q deficiency replacement therapy seems to be the logical solution ^112^. Indeed, hematopoetic stem cell transplantation was recently shown to be successful treatment for hereditary C1q immunodeficiency ^113^. For replacement therapy the administration of C1q or natural IgM is a potential solution. Immunoglobulin preparations for high dose intravenous immunoglobulin therapy contain mainly IgG and exert their effects via pathways related to IgG. However, successful management of SLE with IgM enriched intravenous immunoglobulin (IVIG) has also been reported ^114^, providing support for the beneficial role of nIgM.

It is tempting to speculate that the production of natural IgM, endothelial turnover and integrity and innate clearance are influenced by environmental factors such as nutrition and lifestyle ^115, 116^ or factors produced by the mucosal microbiome, like vitamin K ^117^. If so, we could even look for prevention strategies in light of this hypothesis. The relatively low concordance of SLE in monozygotic twins ^118^ implies that there is ample room for the modulation of environmental effects.

## Concluding remarks and future directions

**Figure 5.**
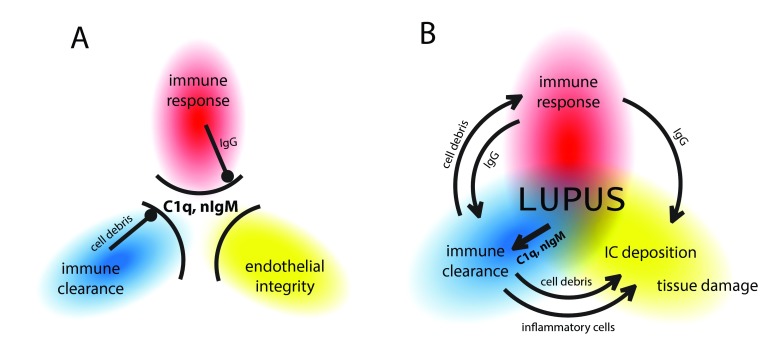
The lupus triangle in health and disease. C1q and natural IgM are gatekeepers that ensure innate immune clearance of apoptotic cellular debris and immune complexes, and also maintain endothelial integrity ( **A**). Under physiological conditions these processes do not interfere with each other. Major abnormality in one or more of these processes, or combinations of minor abnormalities lead to imbalance, consumption of the gatekeepers, the breakdown of these gates and the development of lupus erythematosus ( **B**). Depending on the contribution of these factors lupus will have different colors and shades, which define distinct disease entities or subtypes within such entities.

We propose an interaction scheme for SLE pathogenesis with three key components, each of these contributing to disease development by their mutual interactions ( Figure 5). These components are innate immune clearance, adaptive immune response quality and endothelial integrity. The endothelial deprotection hypothesis assumes that the mechanisms of innate clearance and endothelial integrity share molecules like C1q and nIgM, therefore the two systems can interfere. We suggest that SLE is the net result of an imbalance between these two systems, which is aggravated by the development of autoreactive antibodies, leading to the leakage of immune complexes from the circulation and the triggering of inflammation in the vessel walls and in the tissues.

It will be imperative to create sets and networks of genetic factors that underlie these events and upon that superimpose the protein interactions to create a framework for further interpretation of cellular and immunological processes leading to various forms and manifestations of lupus. We speculate that other systemic autoimmune diseases will share some of these components while also possessing distinct other susceptibility factors to create a continuum of diseases with overlaps. We hope that this hypothesis will serve the further understanding of lupus and these other related diseases as well, leading to novel medical approaches and improvement in the quality of life of all those suffering from these conditions.
