# Supplementary material for: The endothelial deprotection hypothesis for lupus pathogenesis: the dual role of C1q as a mediator of clearance and regulator of endothelial permeability
Source: F1000Res. 2015 May 11;4:24. Originally published 2015 Jan 26. [Version 2] doi: 10.12688/f1000research.6075.2 (PMC4392829; doi:10.12688/f1000research.6075.2)
Supplement: Peer review history

# Peer review: Referee response for version 1

**DOI:** 10.5256/f1000research.6504.r8022

**Published:** 2015-03-18

**Authors:** Fleur Bossi

**Affiliations:** 1 Department of Life Sciences, University of Trieste, Trieste, Italy

The title and Abstract are appropriate.

The article is well written, and the hypothesis is consistent and supported by a good number of references.

The conclusions are balanced and well documented.

Minor comments:

Page 3 Figure 1 endothel should be endothelial

Page 7 Figure 2 suffient should be sufficient

Page 7 Figure 2 Legend (A) endothel should be endothelial

I have read this submission. I believe that I have an appropriate level of expertise to confirm that it is of an acceptable scientific standard.

# Response

**Published:** 2015-04-30

**Authors:** József Prechl

Thank you for reading our paper with scrutiny and helping to improve the quality of the paper. We have made the corrections you suggested in the second version.

# Peer review: Referee response for version 1

**DOI:** 10.5256/f1000research.6504.r7993

**Published:** 2015-03-17

**Authors:** Gregg Silverman

**Affiliations:** 1 Department of Medicine, New York University Langone Medical Center, New York, NY, USA

The manuscript of Prechl and Czirjak provides a thoughtful review of the interface of endothelial cells and soluble factors of complement and antibodies in the pathogenesis of SLE and related diseases. I would recommend to the authors that they also consider the work of two independent groups on the effects of autoantigen specific IgM in the development of post ischemic injury to the vasculature ^1^^,^^2^. Candidate auoantigens have been identified (i.e., annexin IV, non-muscle myosin heavy chain II) ^3^. Overt apoptosis of the endothelial cells may not be an absolute requirement for the expression of modified self antigens that then become accessible to IgM molecules. Furthermore the authors comment that products of complement activation are involved in the clearance of injured and apoptotic cells, but there is extensive evidence that deposition of C1q or MBL are themselves sufficient to serve as “eat me “ signals ^4^^,^^5^. Complexes of (auto) antigen and IgM can therefore enhance phagocytic clearance as they can further enhance MBL and C1q recruitment ^6^.

I have read this submission. I believe that I have an appropriate level of expertise to confirm that it is of an acceptable scientific standard.

## References

[1] KulikLFlemingSDMoratzCReuterJWNovikovAChenKAndrewsKAMarkaryanAQuiggRJSilvermanGJTsokosGCHolersVM: Pathogenic natural antibodies recognizing annexin IV are required to develop intestinal ischemia-reperfusion injury. *J Immunol*.2009;182(9) : 10.4049/jimmunol.08039805363-5373 10.4049/jimmunol.0803980 Reference source 19380783PMC2820395

[2] ZhangMTakahashiKAlicotEMVorup-JensenTKesslerBThielSJenseniusJCEzekowitzRAMooreFDCarrollMC: Activation of the lectin pathway by natural IgM in a model of ischemia/reperfusion injury. *J Immunol*.2006;177(7) : 10.4049/​jimmunol.177.7.47274727-4734 10.4049/jimmunol.177.7.4727 Reference source 16982912

[3] ZhangMAlicotEMCarrollMC: Human natural IgM can induce ischemia/reperfusion injury in a murine intestinal model. *Mol Immunol*.2008;45(15) : 10.1016/j.molimm.2008.06.0134036-4039 10.1016/j.molimm.2008.06.013 Reference source 18672288PMC3230121

[4] NavratilJSWatkinsSCWisnieskiJJAhearnJM: The globular heads of C1q specifically recognize surface blebs of apoptotic vascular endothelial cells. *J Immunol*.2001;166(5) : 10.4049/​jimmunol.166.5.32313231-3239 10.4049/jimmunol.166.5.3231 Reference source 11207277

[5] TaylorPRCarugatiAFadokVACookHTAndrewsMCarrollMCSavillJSHensonPMBottoMWalportMJ: A hierarchical role for classical pathway complement proteins in the clearance of apoptotic cells in vivo. *J Exp Med*.2000;192(3) : 10.1084/jem.192.3.359359-366 10.1084/jem.192.3.359 Reference source 10934224PMC2193213

[6] ChenYParkYBPatelESilvermanGJ: IgM antibodies to apoptosis-associated determinants recruit C1q and enhance dendritic cell phagocytosis of apoptotic cells. *J Immunol*.2009;182(10) : 10.4049/jimmunol.08041916031-6043 10.4049/jimmunol.0804191 Reference source 19414754PMC4428684

# Response

**Published:** 2015-04-30

**Authors:** József Prechl

Thank you for taking the time to read and constructively comment on our hypothesis.  We have incorporated some of the suggested papers as references. Actually we tried to build our hypothesis mostly on human data, because of the known differences in complement regulation between human and mouse. It was of course unaviodable to utilize murine experimental observations, but we kept it to a minimum. Nevertheless, some of the references are murine experimental data and biochemical observations probably hold for the human as well. More caution is probably needed when biological phenomemon and specific immune mechanisms, as specificity of natural antibodies, are considered. A careful comparison and analyis of human and murine data is probably required but this exceeded the scope of this hypothesis paper.

# Peer review: Referee response for version 1

**DOI:** 10.5256/f1000research.6504.r7494

**Published:** 2015-02-23

**Authors:** Marko Radic

**Affiliations:** 1 Microbiology, Immunology and Biochemistry, University of Tennessee Health Science Center, Memphis, TN, USA

Prechl and Czirjak present an attempt to synthesize several aspects of lupus pathogenesis.  In many respects, the opinion article reviews well known territory and the novel aspect is that the authors seek to include a role for complement C1q in the processes of transendothelial transport and paracellular leakage.  These events could be compromised by the reduced levels of C1q that are associated with lupus or with DNA-anti-DNA immune complexes (IC).  The proposed hypothesis is appealing because it may account for the early steps in pathogenesis.  It may also provide a convincing connection to sites of vascular disease and IC deposition.  One aspect that is not thoroughly covered is the deposition of C1q at sites of endothelial discontinuity.  These are presented as areas that arise from the apoptotic death of endothelial cells which could expose underlying ECM.  However, previous reports of apoptosis in a continuous endothelium revealed the rapid displacement of the dead cell by surrounding cells in the cell layer.  This needs to be discussed more fully.  In addition, the primary references describing the role of C1q in endothelial healing and renewal are sparse and incomplete.  Possibly, the involvement of C1q in angiogenesis could be related to the proposed process but the connection is not clearly developed.  Overall, the opinion piece could be streamlined by focusing on the innovative concept of C1q's role in regenerating endothelial gaps. 

Minor comments:

- Figure 1 shows brackets that do not make sense mathematically. Why are genetic factors shown in purple?
- "Endothel" should be endothelium.
- Figure 2 is a bit busy and difficult to deconstruct.  Also the color of arrows is not explained.
- Figure 3 is confusing as to what factors make the three colored ovals merge and overlap.

I have read this submission. I believe that I have an appropriate level of expertise to confirm that it is of an acceptable scientific standard, however I have significant reservations, as outlined above.

# Response

**Published:** 2015-04-30

**Authors:** József Prechl

Thank you for your thorough analysis of our hypothesis and its description. We do agree that the data supporting the role of C1q in regulating endothelial permeability, healing and renewal is sparse. This is main the reason for calling this work a hypothesis and not yet a theory. The reports of C1q influencing endothelial renewal however do fit into the overall emerging big picture of the complement system playing a general role in morphogenesis and tissue repair. We hope and expect to see more experimental data on these aspects of complement in the near future. To highlight the interactions of C1q with the endothelium and the consequences of C1q-containing immune complexes binding to the endothelium we have inserted two new figures into the second revised version of the paper.

The speed of dead endothelial cell replacement and also the turnover rate of these cells are difficult issues. Data are not consistent on these values but because of the huge surface the endothelium covers we expect that even minor and accidental discontinuities count and serve as sites of damage initiation. This may well contribute to the randomness of the development of damage sites and of flares.

We accept that brackets and parentheses do not change the result of a multiplication when numbers are multiplied. If we consider these factors not as numbers but something more complex, like matrices, then brackets might make sense. Here, using the brackets and blue/purple color, we only intended to highlight that genetic variability mainly affects proteins contributing to physiology and immunity but not anatomy. We explained this in the legends now.

Syntax errors were corrected as suggested.

We explained the colors of the arrows in figure 2. To make this figure somewhat less busy we also decreased the size of the arrows in between the boxes.

We think that it is one major abnormality (e.g. C1q deficiency) or the combination of several abnormalities (multiple genetic factors affecting e.g. apoptotic debris clearance via CR3, adaptive response e.g. via HLA alleles, together with environmental effects, like an infection) that trigger the imbalance and the merging of these different pathways. The consumption and relative depletion of C1q and natural IgM is key to this overlap and lupus development.

To help the reader follow this line of thinking we added „consumption of the gatekeepers,” to the legend.

We hope that our responses and corrections are acceptable and improve understandability.
